# *QuickStats*: Percentage* of Adults Aged ≥18 Years with Diagnosed Heart Disease,^†^ by Urbanization Level^§^ and Age Group — National Health Interview Survey, United States, 2020^¶^

**DOI:** 10.15585/mmwr.mm7123a4

**Published:** 2022-06-10

**Figure:**
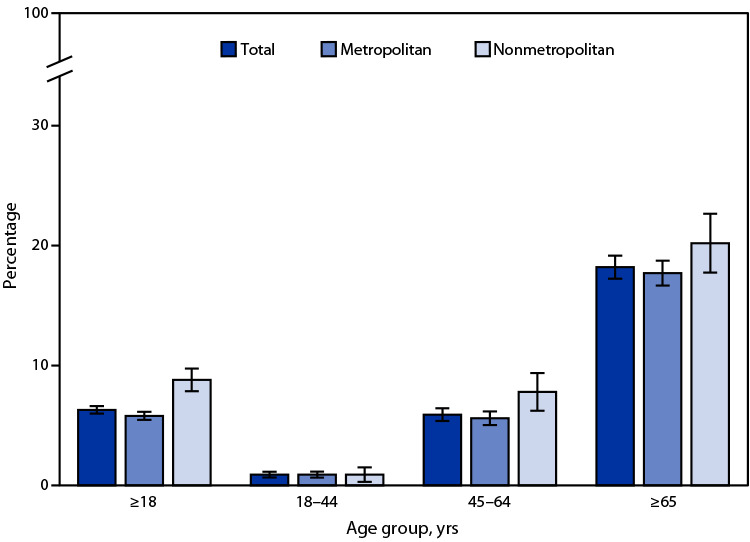
In 2020, 6.3% percent of adults aged ≥18 years had diagnosed heart disease. The prevalence of heart disease among adults aged ≥18 years was higher among those living in nonmetropolitan areas (8.8%) compared with those living in metropolitan areas (5.8%). Prevalence increased with age from 0.9% among adults aged 18–44 years to 5.9% among those aged 45–64 years and 18.2% among those aged ≥65 years. Among adults aged 45–64 years, those living in nonmetropolitan areas (7.8%) were more likely to have heart disease than those living in metropolitan areas (5.6%). There was no statistically significant difference by urbanization level for adults aged 18–44 or ≥65 years.

**Source**: National Center for Health Statistics, National Health Interview Survey, 2020. https://www.cdc.gov/nchs/nhis.htm

